# MitoScore, MitoGrade, or MitoSure: what does embryonic mitochondrial deoxyribonucleic acid quantification actually measure and is it useful?

**DOI:** 10.1016/j.xfre.2022.02.001

**Published:** 2022-02-05

**Authors:** Richard J. Paulson

**Affiliations:** Division of Reproductive Endocrinology & Infertility, Department of Obstetrics & Gynecology, Keck School of Medicine, University of Southern California, Los Angeles, California

One of the keys to maximizing the efficiency of in vitro fertilization is the determination of which embryos have the greatest likelihood of implantation. This is the purpose of morphological assessment, time-lapse evaluation of embryonic growth, and even preimplantation genetic testing for aneuploidy (PGT-A). Recent reports have suggested that mitochondrial deoxyribonucleic acid (mtDNA) quantification has good predictive value for implantation potential (1, 2). In one of these reports, the investigators actually identified a cutoff value of mtDNA that predicted 0% implantations (2). This is a remarkable finding, but what is even more remarkable is that *higher* mtDNA quantities were found to be associated with *lower* implantation potential. These findings seem to contradict the previously reported observation that a *higher* metabolic activity of the embryo is correlated with *better* implantation potential (3). The latter observation seems logical if we accept the premise that embryos that develop faster are more likely to have a higher implantation potential and greater metabolic activity. If a higher metabolic activity is associated with higher implantation rates and if mitochondria are responsible for metabolism, then we should expect that *more*, rather than *less*, mitochondria (as measured by mtDNA quantification) should be beneficial. Additionally, the high variability in mtDNA measurements appears to contradict the observation that mitochondrial replication does not begin until after implantation (4). How can it be that the mtDNA content in embryos is so variable, and why does it seem that “less is better” (1)?

To understand how mtDNA measurements are reported, we have to delve into the materials and methods sections of the reports (1, 2). Because DNA measurements are made on trophectoderm biopsies, the investigators needed some sort of estimate of how large the biopsy was to compare DNA content between embryos. Reports of mtDNA quantification “normalize” the mtDNA content to nuclear DNA (nDNA) content (1, 2), meaning that the reported mtDNA measurements do not represent the quantitative mtDNA copy number but rather the ratio of mtDNA to nDNA. Because the value is a ratio, both the numerator and denominator may influence the final value. In this case, the denominator is the quantity of nDNA, which is one way of estimating how much of the embryo was removed in the biopsy. However, more precisely, nDNA quantification is a measure of how many *nuclei* were removed in the biopsy because each nucleus typically contains 1 copy of nDNA. Because most trophectoderm cells contain 1 nucleus and the size of individual cells varies, the nDNA measurement must vary with the size of the individual cells, and cell size will necessarily influence the final mtDNA/nDNA ratio.

During a trophectoderm biopsy, nuclei and individual cells are not visible. The embryologist performing the biopsy can control the total amount of cytoplasm that is removed from the embryo but can only estimate the total number of cells in the biopsy. An embryo that is developing faster contains more (smaller) cells, and one that is developing slower contains fewer (larger) cells. Biopsies of the same size in different embryos, therefore, contain different numbers of cells and different numbers of copies of nDNA. One biopsy may contain 5 cells, whereas the same sized biopsy in a blastocyst with smaller blastomeres may contain 10 cells. Even if the total size of the biopsy is the same and the mtDNA content is the same, the ratio of mtDNA to nDNA will nevertheless, be twice as high in the biopsy with 5 cells, rather than 10 cells. Embryos with a faster rate of cell cleavage have smaller cells, more copies of nDNA, and a lower mtDNA/nDNA ratio. Conversely, a slower rate of cell division will result in larger trophectoderm cells and a higher mtDNA/nDNA ratio. Therefore, the findings that a higher metabolic activity (3) and smaller mtDNA/nDNA ratio (1, 2) are both associated with a higher implantation potential are consistent.

The confusion stems from the designation of the mtDNA/nDNA ratio as “mitochondrial DNA content” (1) or “mitochondrial DNA quantification” (2), which ignores the fact that this is a ratio, in which the denominator (nDNA) can vary widely. Because mitochondrial replication does not begin until after implantation (4), increases in the mtDNA/nDNA ratio are more likely to be due to blastomere size than to an increase in the number of mtDNA copies.

This analysis is not intended as a criticism of the current practice of mtDNA quantification. If the mtDNA/nDNA ratio is actually an indirect assessment of the size of the blastomeres, it has the potential to be a very good marker of the developmental stage of the blastocyst. Larger numbers of the ratio represent smaller quantities of nDNA, larger trophectoderm cells, slower development, and lower implantation potential. The report of 0 implantations in embryos with mtDNA/nDNA ratios above a certain threshold (2) may well be an observation that a certain minimum number of cells are required for a blastocyst to achieve implantation. If this is true, it may provide an important physiologic insight. It may also be an indication that a certain minimum metabolic activity is required to accomplish implantation and endometrial invasion. Since the mtDNA/nDNA measurement is part of many PGT-A reports, the value may be specifically helpful in deciding which euploid blastocyst to transfer first.

The appreciation of the role that blastomere size must play in reported mtDNA measurements may help quell some of the controversy that surrounds mtDNA assessments, recently reviewed by Cecchino and Garcia-Velasco (5). In the meantime, we should acknowledge that the reported mtDNA measurements do not quantify the total mtDNA content of an embryo. We should also call the measurements what they actually are: ratios, not determinations of absolute quantity or content. Instead, of MitoScore, MitoGrade, or MitoSure, why not consider “Mito-Nuclear Ratio” as a more precise description of this measure?
